# A preoperative simulation of medial open-wedge high tibial osteotomy for predicting postoperative realignment

**DOI:** 10.3389/fbioe.2023.1278912

**Published:** 2023-12-21

**Authors:** Shoji Konda, Teruya Ishibashi, Masashi Tamaki, Tetsuya Tomita

**Affiliations:** ^1^ Department of Health and Sport Sciences, Graduate School of Medicine, Osaka University, Toyonaka, Japan; ^2^ Department of Orthopaedic Biomaterial Science, Graduate School of Medicine, Osaka University, Suita, Japan; ^3^ Department of Orthopaedic Surgery, Graduate School of Medicine, Osaka University, Suita, Japan; ^4^ Graduate School of Health Sciences, Morinomiya University of Medical Sciences, Osaka, Japan

**Keywords:** knee, osteoarthritis, medial OWHTO, preoperative surgical simulation, hinge axis

## Abstract

Three-dimensional preoperative surgical simulation of the medial open-wedge high tibial osteotomy (OWHTO), simplified as the rigid rotation around the hinge axis, has been performed to predict postoperative realignment. However, the practicality of this highly simplified simulation method has not been verified. This study aimed to investigate the validity of realignment simulation simplified as a rotation around a hinge axis compared with a postoperative CT model. A three-dimensional surface model of the tibia and femur was created from preoperative computed tomography (CT) images (preoperative model) of three patients. The simulation of medial OWHTO created sixty computer simulation models in each patient simplified as the rigid rotation of the proximal part of the tibia relative to the distal part from 1° to 20° around three types of hinge axes. The simulation models were compared with the actual postoperative model created from postoperative CT images to assess the reality of the simulation model. The average surface distance between the two models was calculated as an index representing the similarity of the simulation model to the postoperative model. The minimum value of average surface distances between the simulation and postoperative CT models was almost 1 mm in each patient. The rotation angles at which the minimum value of average surface distances was represented were almost identical to the actual correction angles. We found that the posterior tibial tilt and the axial rotation of the proximal tibia of the simulation model well represented those of the postoperative CT model, as well as the valgus correction. Therefore, the realignment simulation of medial OWHTO can generate realistic candidates for postoperative realignment that includes the actual postoperative realignment, suggesting the efficacy of the preoperative simulation method.

## Introduction

The medial open-wedge high tibial osteotomy (OWHTO) aims to realign the femur and tibia (Lobenhoffer and Agneskirchner, 2003). The medial OWHTO corrects the varus deformity by shifting the weight-bearing axis from the medial compartment to the lateral compartment (valgus correction), thereby reducing the excessive load on the medial compartment (Agneskirchner et al., 2007). In addition to valgus correction, control of the posterior tibial slope (PTS) and internal/external rotation are crucial factors associated with the postoperative result (Jang et al., 2016; Lee et al., 2017). Three-dimensional (3D) preoperative surgical realignment simulation of medial OWHTO has been performed to predict the postoperative change in the PTS and internal/external rotation as well as the valgus correction (Song et al., 2007; Chernchujit et al., 2019; Kang et al., 2022). In addition, 3D preoperative surgical realignment simulation has been used to develop a patient-specific instrument for accurate osteotomy (Victor and Premanathan, 2013; Donnez et al., 2018; Jörgens et al., 2022). Muscle skeletal simulation has been performed using a postoperative realignment simulation model to predict the change in the intra-articular load after medial OWHTO (Kuriyama et al., 2019; Kuriyama et al., 2020b).

The postoperative realignment simulation of medial OWHTO was performed using preoperative computed tomography (CT) images and a 3D model. In the postoperative realignment simulation of medial OWHTO, the position and orientation of the hinge axis and the osteotomy plane through that axis are defined, and the postoperative realignment is predicted by cutting the 3D model at the osteotomy plane and realigning it around the hinge axis (Moon et al., 2015; Lee et al., 2017; Teng et al., 2021; Jörgens et al., 2022; Thürig et al., 2022). Combining the hinge axis and the rotation angle around the axis makes it possible to create various postoperative simulation models systematically. The simplified simulation has been verified for the preoperative simulation of the upper extremity (Murase et al., 2008; Murase, 2018). However, the realistic practicality of this highly simplified simulation model of a rotation axis and rotation around that axis has not been verified for OWHTO. To verify whether the virtual realignment generation realistically reflects the postoperative realignment, it is necessary to compare it with the actual postoperative realignment. Therefore, this study aimed to investigate the validity of realignment simulation, in which medial OWHTO is simplified as a rotation around a hinge axis, compared with a postoperative CT model.

## Materials and methods

**FIGURE 1:**
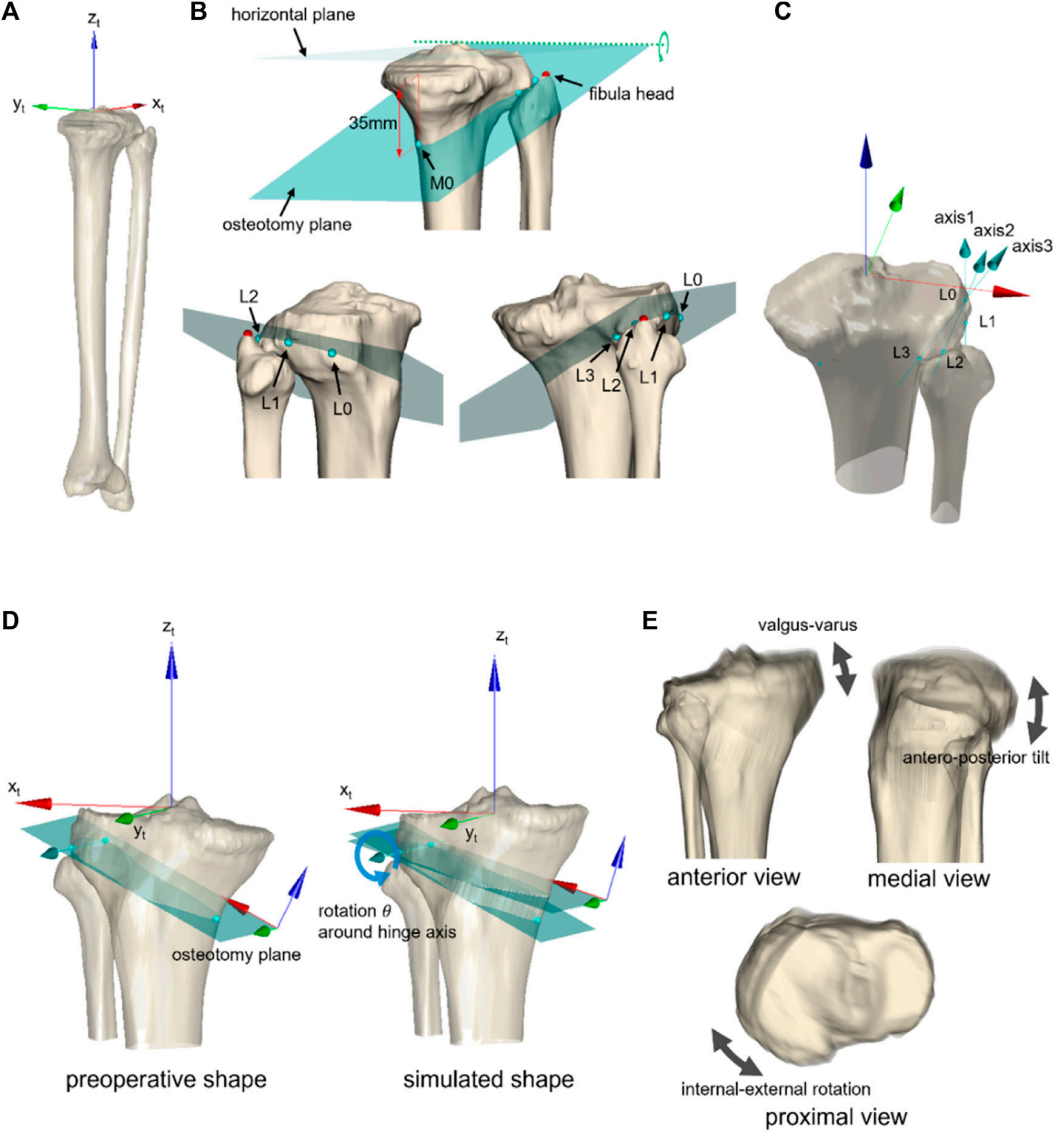
Definition of bony landmarks and axis of rotation for realignment simulation. **(A)** A preoperative 3D surface model was developed using CT images, and the preoperative CT-driven model was spatially normalized by the anatomical coordinate system. **(B)** Cutting plane was defined by rotating the horizontal plane around the *y*-axis passing the fibula head and M0 (35 mm inferior to the medial edge of the articular surface). Additional landmarks (L0, L1, L2, and L3) were identified along with the cutting plane for simulating variation of clinically observed hinge axes in the medial OWHTO **(C)**. **(D)** Simulation of medial OWHTO rotating the proximal tibia around the given axis of rotation and **(E)** variation of 60 simulated shapes (3 axes × 20 rotation angles) of the proximal tibia.

The study was conducted in accordance with the guidelines of the Declaration of Helsinki and approved by the Institutional Ethics Committee of Osaka University Hospital (19027-2). All participants provided written informed consent. A male patient (75 years, 167.0 cm, 69 kg) and two female patients (71 years 151.2 cm, 50.8 kg and 69 yrs 146 cm, 72 kg) who were scheduled for medial OWHTO were included in this study. CT images of the three patients were acquired using a 3D-CT imaging system (Optima CT660Pro Advance; GE Healthcare, Milwaukee, WI), under the following conditions: slice thickness (1.25 mm), tube voltage (120 kV), tube current (440 mA), and acquisition matrix (512 × 512). The contours of the cortical bone in the tibia, fibula, and femur were semi-automatically segmented from the CT images using a 3D image analysis workstation (Volume Analyzer Synapse Vincent, Fujifilm Corporation, Tokyo, Japan). A 3D surface model of the bones was reconstructed from the segmented images (preoperative CT model) (Figure 1A).

Three anatomical landmarks of the tibia (insertion of the anterior cruciate ligament and posterior cruciate ligament, and the center of the distal articular surface) were identified on the preoperative CT model to define the tibial anatomical coordinate system (Tajima et al., 2009; Kusano et al., 2017). The preoperative CT model of the tibia and femur was transformed into the tibial coordinate system for spatial normalization. Five additional landmarks were identified to determine the cutting plane (Figure 1B), and three axes of rotation (hinge axes) were defined (Figure 1C). The variations in the hinge axis can represent the clinically observed type of osteotomy (Ogawa et al., 2017). The variations are set within the “safe zone” of medial OWHTO (Han et al., 2013). Vertices of the preoperative CT model located above the osteotomy plane were translated by the matrix transformation (Figure 1D), which was determined by the rotation angle ( θ ) around the hinge axis. In total, 60 simulations of the medial OWHTO in each patient were obtained with a combination of the 20 rotation angles and three hinge axes. The proximal part of the tibia of the simulation models showed anteroposterior tilting and internal-external rotation in addition to valgus-varus rotation because the given axes of rotation were not along the anteroposterior axis of the tibia (Figure 1E).

**FIGURE 2:**
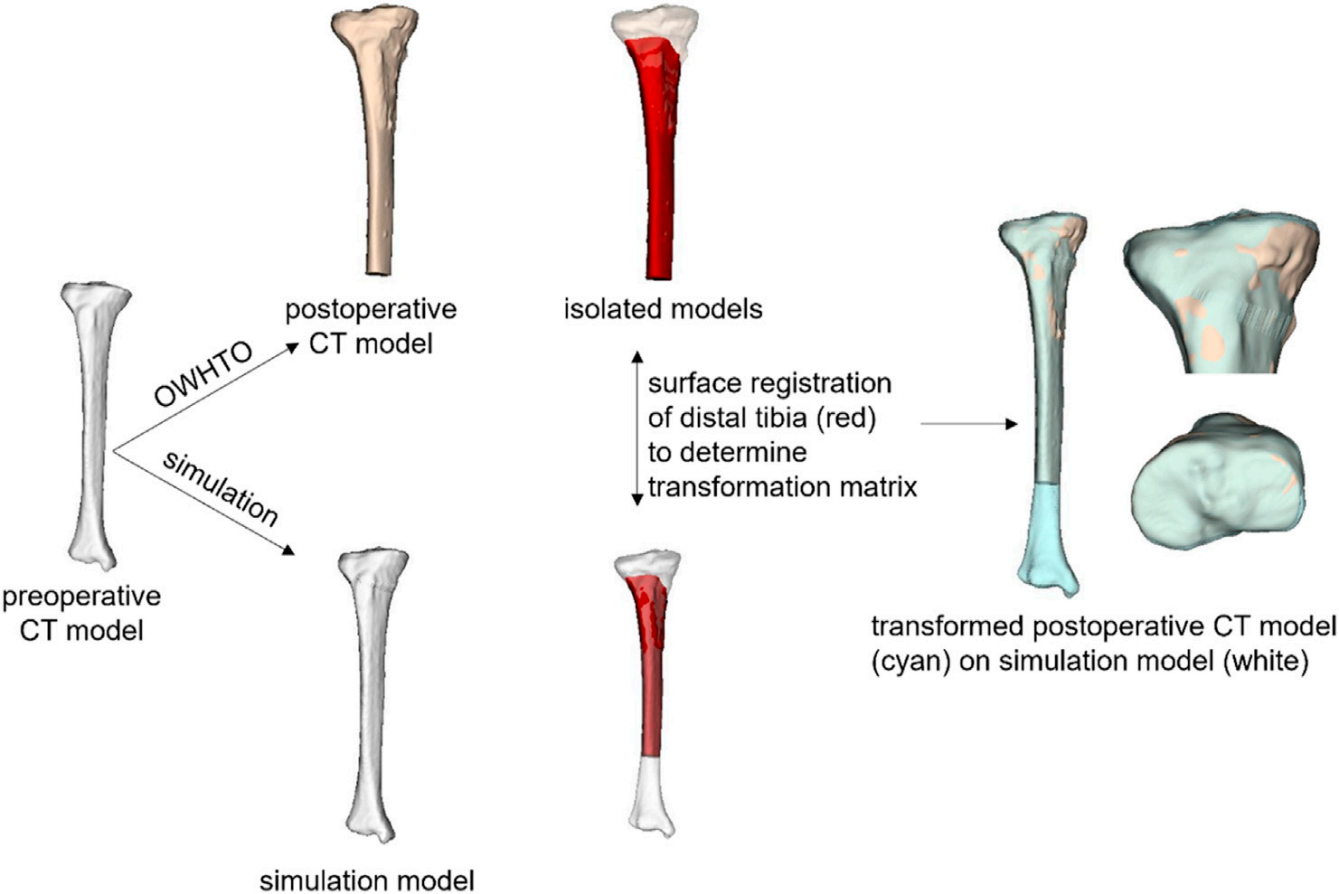
Comparison of simulation model and postoperative CT model. After the simulation and postoperative CT models were aligned by a surface registration of the distal part of the tibia, the average surface distance between the two models was calculated as an index representing the similarity of the simulation model to the postoperative model.

The simulation models were compared with the actual postoperative model to assess the reality of the simulation of medial OWHTO simplified as the rotation around the hinge axis. The postoperative tibial model was created with a semiautomatic segmentation from the postoperative CT images. The inserted metal fixation plate was deleted manually. The postoperative CT model was cut below the osteotomy plane, where the shape was not changed before and after medial OWHTO. Surface registration of the distal part of the tibia was performed to align the simulation and postoperative CT models using the Image Registration Tool Kit (Rueckert et al., 1999). Afterward, the surface distance of the proximal tibia between the simulation models and postoperative CT models was evaluated. The average surface distance was calculated between each simulation model and postoperative CT model as an index representing the similarity of the simulation model to the postoperative model (Figure 2).

## Results

**FIGURE 3:**
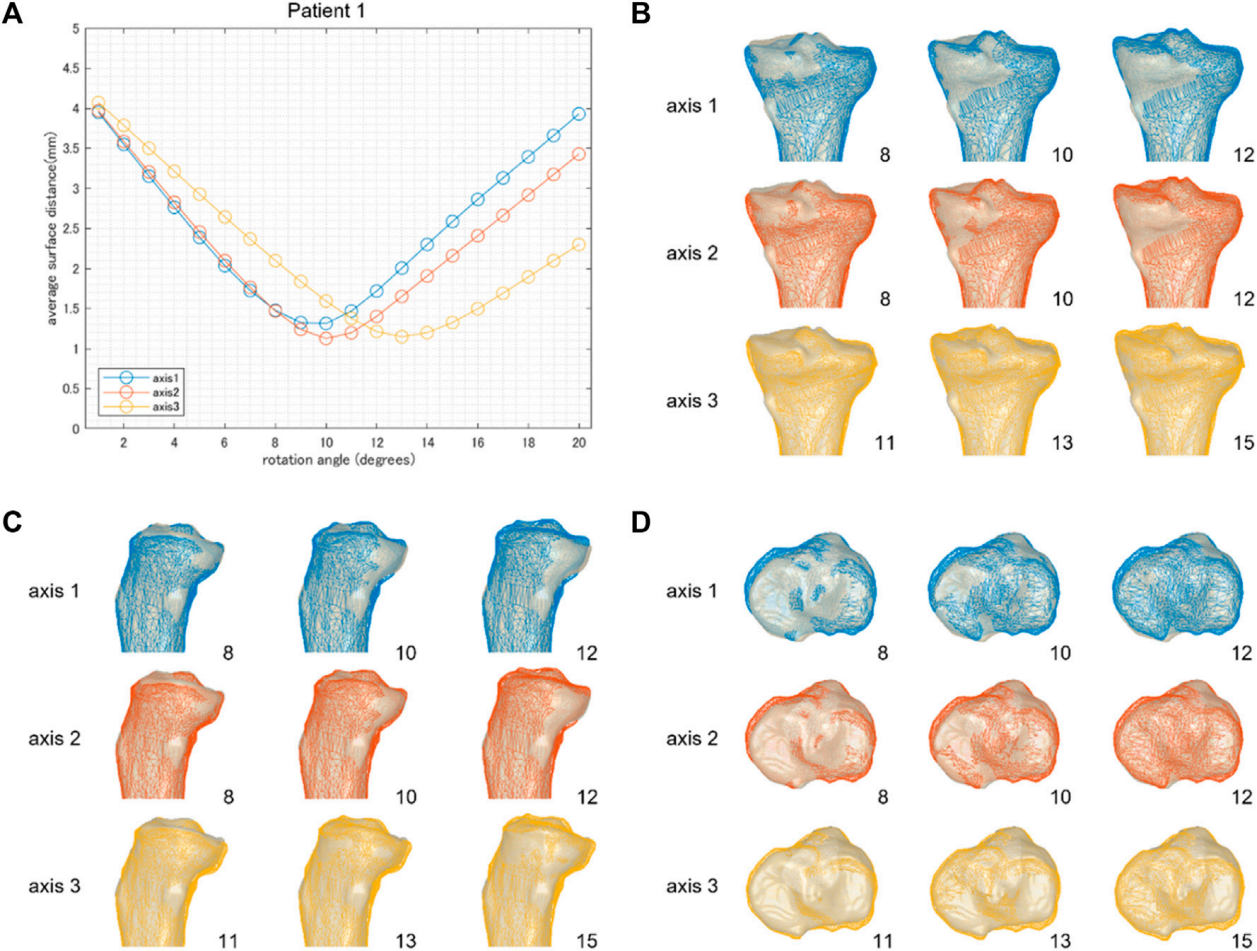
The results of patient 1 who underwent an average correction angle (10.3° by the postoperative x-ray evaluation). **(A)** The average surface distance of each simulation model, generated by the combination of hinge axis and rotation angle, relative to the postoperative CT model. **(B–D)** Backward, medial, and proximal viewed images of overlay of the simulation model generated by three different hinge axes (wire model in colors) on the postoperative CT model. The number on the bottom right shows the rotation angle around each hinge axis.

**FIGURE 4:**
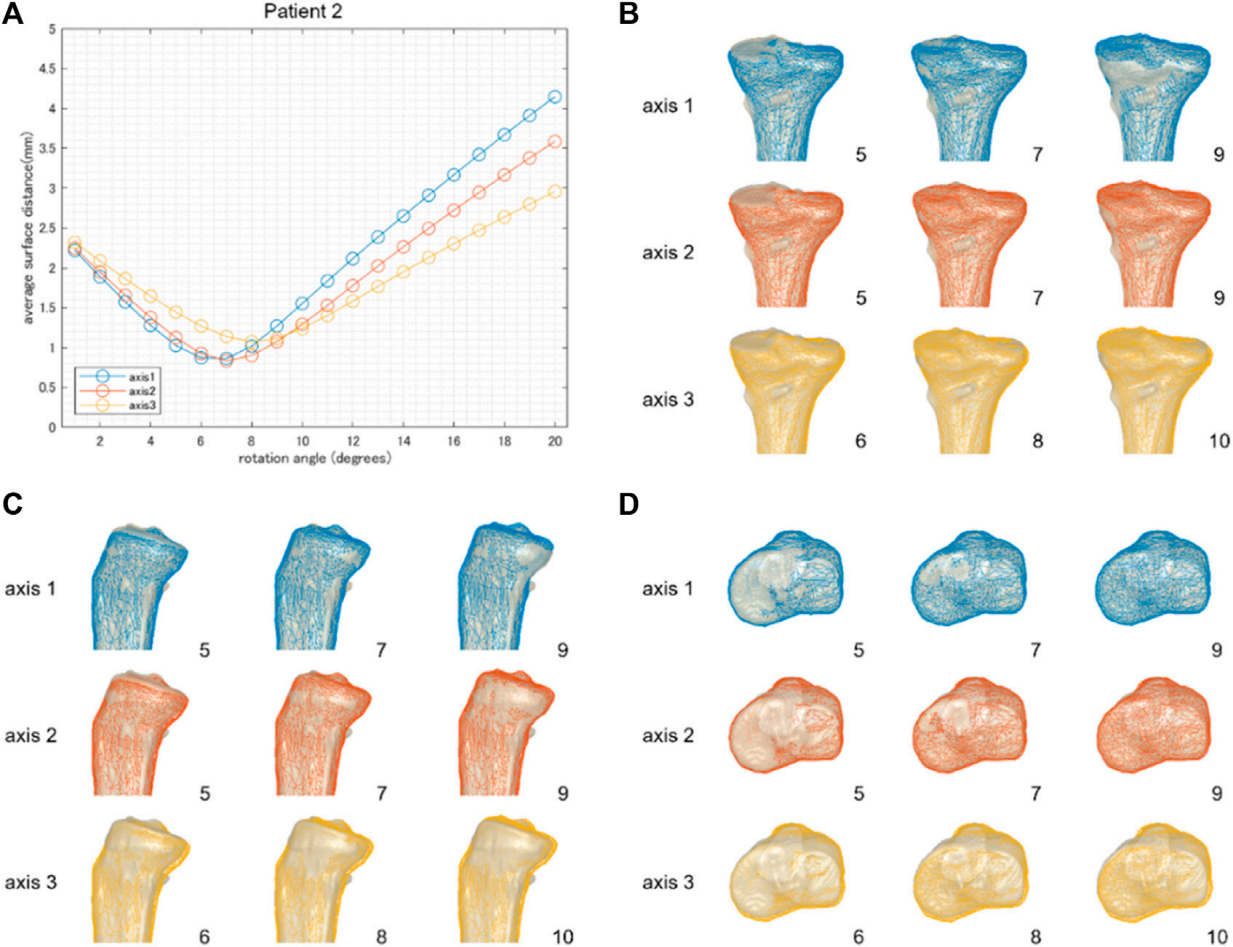
The result of patient 2 underwent a relatively small correction angle by medial OWHTO (8.2° by postoperative x-ray evaluation). The average surface distance was smallest when rotated by 7 or 8° **(A)**. The rotation angle was consistent with the postoperative correction angle recorded by the postoperative X-ray evaluation. The simulation model (wire model in colors) representing the smallest surface distance in each hinge axis (center column) demonstrated that the valgus angle of the tibial plane is more consistent with the postoperative CT model on the backward view **(B)**. When compared within the model in the center column, the models generated by axis 1 and axis 2 were more fitted to the postoperative CT model than axis 3 in the posterior tibial slope **(C)** and axial rotation of the tibial plane **(D)**. The number on the bottom right shows the rotation angle around each hinge axis.

**FIGURE 5:**
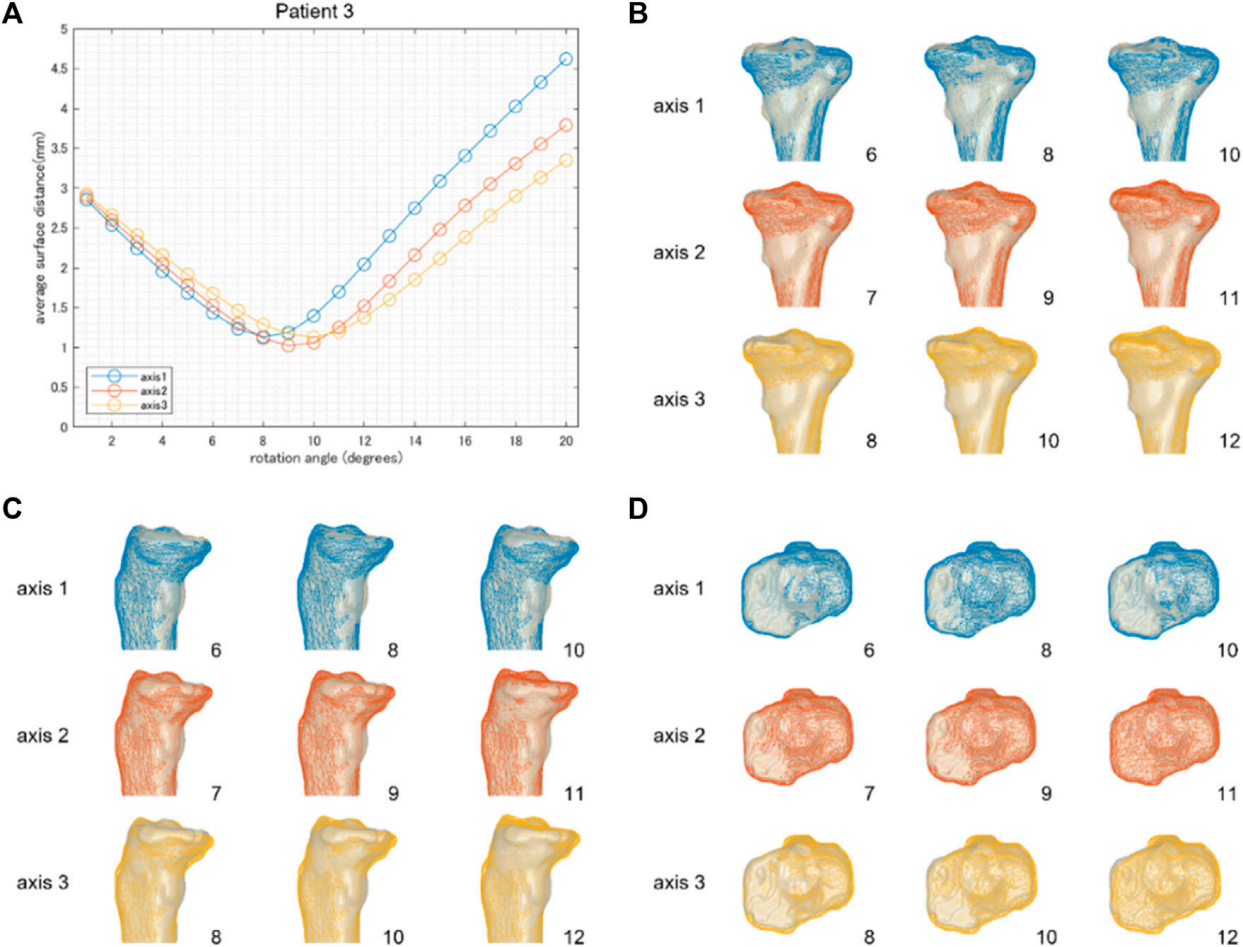
The results of patient 3 underwent a relatively large correction angle by medial OWHTO (12.2° by postoperative x-ray evaluation). The average surface distance was smallest when it was rotated by 8, 9, and 10° around hinge axes 1, 2, and 3, respectively **(A)**. The rotation angle was inconsistent with the postoperative correction angle recorded by the postoperative X-ray evaluation. The simulation model (wire model in colors) representing the smallest surface distance in each hinge axis (center column) demonstrated that the valgus angle of the tibial plane is more consistent with the postoperative CT model on the backward view **(B)**. When compared within the model in the center column, the model generated by axis 2 was more fitted to the postoperative CT model than axis 1 and axis 3 in the posterior tibial slope **(C)** and axial rotation of the tibial plane **(D)**. The number on the bottom right shows the rotation angle around each hinge axis.

Figure 3 demonstrates the results of patient 1, who underwent an average correction angle by medial OWHTO (10.3° by the postoperative X-ray evaluation). When the axis of rotation was set to axis 1 or axis 2, the average surface distance was smallest when it was rotated by 10°. The rotation angle was consistent with the postoperative correction angle recorded by the postoperative X-ray evaluation. On the other hand, when the axis of rotation was set to axis 3, the average surface distance was smallest when it was rotated by 13° (Figure 3A). Graphics show the overlay of the simulation model generated by three different hinge axes (wire model in colors) on the postoperative CT model. The rotation angle of the model displayed in the graphics was set to the angle with the smallest surface distance, and −2° and +2° to it. The simulation model representing the smallest surface distance in each hinge axis (center column) demonstrated that the valgus angle of the tibial plane was more consistent with the postoperative CT model on the backward view (Figure 3B). The simulation model of −2° and +2° demonstrated the under (left column) and overcorrection (right column) of medial OWHTO (Figure 3B). When compared within the model in the center column, the model generated by axis 2 was more fitted to the postoperative CT model than axis 1 and axis 3 in the posterior tibial slope (Figure 3C). The models generated by axis 2 or axis 3 were more fitted to the postoperative CT model than axis 1 in the axial rotation of the tibial plane (Figure 3D). The simulation model could also represent the postoperative CT model in patients 2 and 3 who underwent relatively small and large correction angles (Figures 4, 5).

## Discussion

We aimed to investigate the validity of the realignment simulation of the medial OWHTO simplified as the rigid rotation around the hinge axis compared to a postoperative CT model. The minimum average surface distance between the simulation model generated by the rotation around three hinge axes and the postoperative CT model was almost 1 mm. The rotation angle at which the minimum average surface distance was represented on each hinge axis was almost identical to the actual correction angle. Overlaying the 3D surface models of the simulation and the postoperative CT, we found that the posterior tibial tilt (Figures 3, 4, 5) and the axial rotation of the proximal tibia (Figures 3, 4, 5) of the simulation model well represented that of the postoperative CT model as well as the valgus correction (Figures 3, 4, 5). Therefore, the realignment simulation of medial OWHTO simplified as the rigid rotation around the hinge axis can generate realistic candidates of postoperative realignment that include the actual postoperative realignment, suggesting the efficacy of the preoperative simulation method.

**FIGURE 6:**
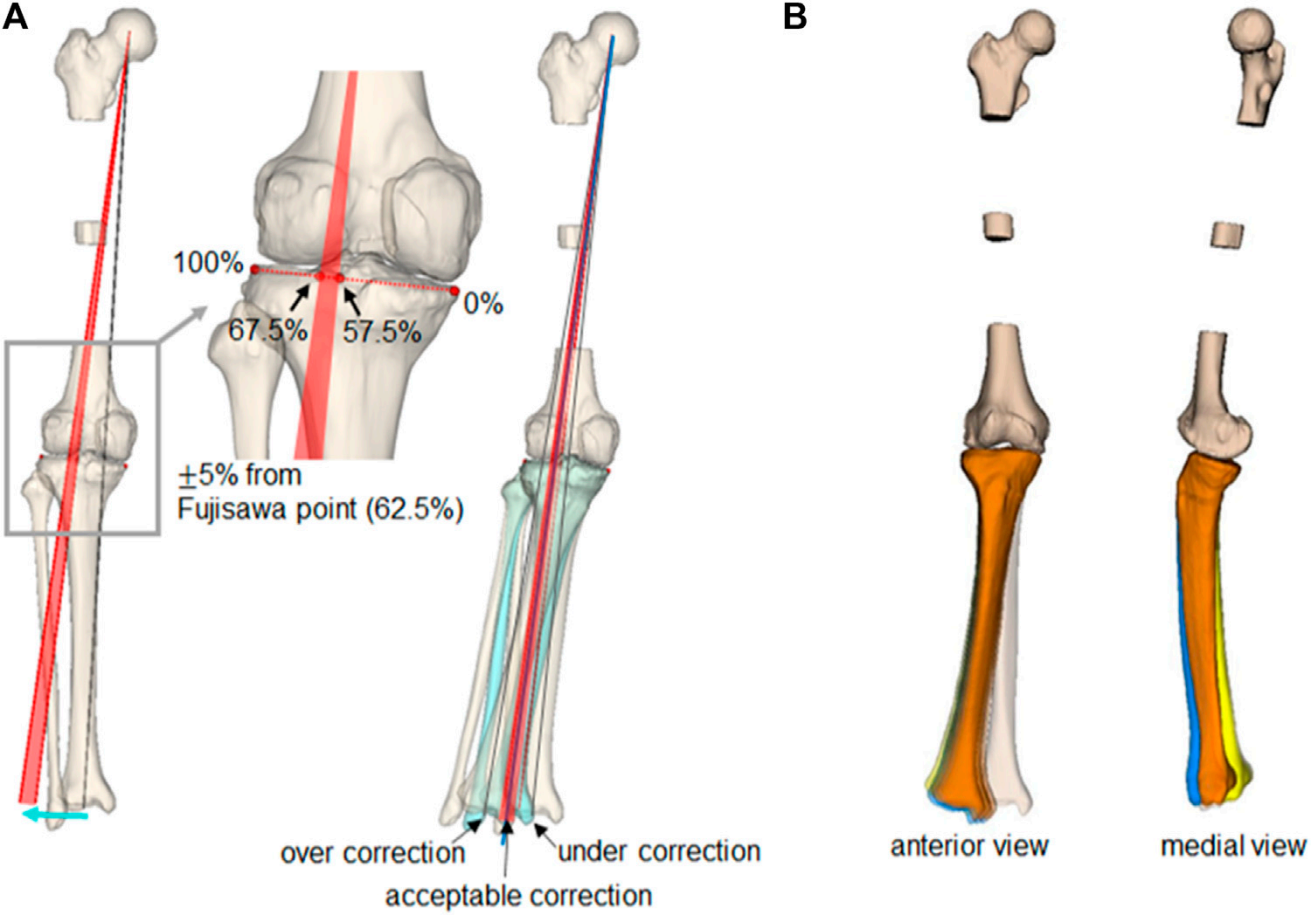
Selection of clinically acceptable simulations and simulated postoperative realignment. **(A)** The gray line shows the preoperative mechanical line, and the red arc shows the clinically acceptable target alignment after the medial OWHTO. The simulation models in which the mechanical line passes within the arc were selected as the clinically acceptable simulation. **(B)** Three-dimensional realignment variation of clinically acceptable simulation models with three types of hinge axes (axis 1: blue, axis 2: orange, axis 3: yellow).

A significant advantage of realignment simulation in which medial OWHTO is simplified as a hinge axis and rotation around that axis is a systematic generation of the candidates of the postoperative realignment. The 60 generated simulation models have great variability; however, some of the simulation models may show an unrealistic realignment. The acceptable simulations in the 60 simulations were selected based on the clinical criteria for femorotibial alignment, as shown in Figure 6A. The medial and lateral edges of the articular surface of the proximal tibia were identified on the frontal view of the simulation model and were defined as 0% and 100%, respectively. A femorotibial alignment where the mechanical line (the line connecting the center of the hip joint center and the center of the distal tibia) passes through 62.5%, called the Fujisawa point, has been recognized as a clinically ideal alignment (Fujisawa et al., 1979). In this study, the acceptable range was set at ± 5% (57.5%–67.5%) from the Fujisawa point (Miniaci et al., 1989; Dugdale et al., 1992; Kuriyama et al., 2020a). The arc passing through points 57.5% and 67.5% centered on the hip joint center were determined to evaluate passes of the mechanical line of the simulation models. The simulation models that passed within the arc were selected, suggesting candidates for clinically feasible postoperative realignment (Figure 6B). An additional advantage is that the simulation can generate variation in postoperative realignment from the preoperative alignment of a single patient. A large-scale database of virtual postoperative realignment can be developed when applied to many preoperative patient alignments, and it will contribute to the virtual installation of the fixation plate in preoperative planning and the development of new surgical devices.

A disadvantage of this simulation method was that the simplified simulation ignored the bone deformability and the biplane medial OWHTO process. The simulation model was created by rigid rotation around the set hinge axis between the proximal and distal tibial parts. In actual intraoperative situations, the tibia can be deformed manually at the preserved cortex when inserting a fixation plate in addition to rotation around the hinge axis. The vertices of the surface model located superior to the osteotomy plane were transformed along the rotation around the hinge axis; however, the tuberosity is preserved with the distal tibial parts in the biplane medial OWHTO. Although the present simulation models the medial OWHTO as the rigid rotation around the hinge axis, the simulation model well represents the postoperative CT model.

A limitation of this study is the small sample size, which may affect the generalizability of our findings. However, it is important to note that, as this study primarily aimed to propose a methodological approach, the small sample size does not fundamentally impact the validity of our results. Further studies with larger sample sizes may be needed to validate our results and to explore the full potential and limitations of the realignment simulation method in the medial OWHTO.

## Conclusion

The realignment simulation of the medial OWHTO simplified as the rigid rotation around the hinge axis can generate realistic candidates of postoperative realignment that include the actual postoperative realignment, suggesting the efficacy of the preoperative simulation method. We believe the simplified realignment simulation can be used for preoperative planning of the medial OWHTO.

## Data Availability

The raw data supporting the conclusions of this article will be made available by the authors, without undue reservation.
